# Staff’s experiences of implementing patient-initiated brief admission for adolescents from the perspective of epistemic (in)justice

**DOI:** 10.3389/fpsyt.2022.1054028

**Published:** 2022-12-15

**Authors:** Jennie Moberg, Ulla-Karin Schön

**Affiliations:** Department of Social Work, Stockholm University, Stockholm, Sweden

**Keywords:** agency, epistemic injustice, recovery, implementation, power, patient-initiated brief admission, child and adolescent mental health care, participation

## Abstract

**Background:**

The implementation of Patient-Initiated Brief Admission (PIBA) in child and adolescent psychiatry (CAP) in Sweden is ongoing. This intervention enables adolescents between the ages of 13–17 and with complex mental health problems to initiate a short care period for relief and support rather than the care apparatus being controlling in this process. Offering it is likely to promote epistemic agency, an exchange of knowledge and recovery from mental health problems.

**Aim:**

The aim of this study was to explore staff’s perspectives of PIBA for adolescents with complex mental health problems, and what facilitates or hinders its implementation.

**Methods:**

Twenty seven employees, 21 women and six men, with various professions in CAP were interviewed and the material was analyzed thematically.

**Results:**

Two overall themes emerged: “Staff’s Experiences of PIBA” and “Managing Clinical PIBA Work.” The results were discussed in relation to the theoretical frameworks of epistemic injustice and Normalization Process Theory (NPT). The main findings indicate that PIBA was generally viewed in a positive way, but that obstacles arose when it was actually put into practice. Findings also point at an overall lack of agency among staff when implementing this new way of working, at the same time as the need to adapt PIBA from an adult psychiatric intervention to one for adolescents in CAP is addressed.

**Conclusion:**

This article offers insights into the views of psychiatric staff regarding the implementation of PIBA. If staff wish to support epistemic agency and recovery among adolescents, their agency may be an important aspect in the continued implementation. Furthermore, in order for PIBA to become normalized in a sustainable way, we suggest that the continued implementation should be characterized by a youth-friendly framework.

## Introduction

In recent years, interest in young people’s agency and position in health care and other welfare services has increased internationally (1, 2). A similar focus can be seen in Sweden, which has also led to a number of legal changes *via*, for instance, the incorporation of the Convention on the Rights of the Child (3) into Swedish law in 2020. This enhanced focus has also contributed to changes in knowledge asymmetries and the Swedish compulsory psychiatric legislation where the child’s position and opportunities for increased rights have been described as important prerequisites for dignified and safe care (4). Giving young people with complex mental health problems increased agency and influence over their care is a task that in many respects requires delicate handling, while it places a great responsibility on the professionals [cf. (5)]. At the same time, promoting recovery is a central perspective in psychiatry which includes aspects of symptom management, participation, hope, meaningfulness, and autonomy (6). However, some patient groups return to psychiatric inpatient care where admissions may be protracted and risk being characterized by coercion and ineffective treatment (7). Also, these patients are more exposed to epistemic violations than others (8–10), which generally complicates agency and recovery. With the aim of improving young people’s agency in psychiatric care, Patient-Initiated Brief Admission (PIBA) has been introduced in Child and Adolescent Psychiatry (CAP) in Stockholm. In summary, PIBA is a standardized crisis management intervention (11) drawing on increased autonomy for patients to self-assess if they require a briefer period of inpatient care, rather than the care apparatus being controlling in this process. One major difference compared to traditional admission is that it is nurses and not doctors that handle the enrollment, but also that no professional assessment is made concerning whether the admission is justified or not—this is determined entirely by the individual.

The adolescent signs an agreement together with his/her parents and caregivers from both outpatient and inpatient care that gives them the opportunity to initiate, regardless of the time of day, a care period of a maximum of four days three times per month. Since inpatient care can be perceived as difficult to access, PIBA has the potential to reduce the struggle for admission that sometimes occur between patients and care staff. Instead, through increased agency, PIBA may simultaneously expand the patient’s interpretative precedence regarding the need for admission, making room for subjective needs and wishes for inpatient care. Through this (tentative) approach, we suggest that there is an explicit idea that PIBA may promote recovery and epistemic justice also for young people since they, to a greater degree, have the possibility to define and voice their needs rather than others defining and voicing these for them [cf. (12, 13)]. Providing PIBA to certain adolescents in CAP may change traditional structures of power and knowledge legitimacy. However, knowledge of its effects is so far limited.

The article explores staff’s perspectives of PIBA for adolescents with complex mental health problems, and what facilitates or hinders its implementation. Two research questions have guided this purpose. (1) How do the staff understand their work with PIBA? and (2) What experiences do the staff have of implementing PIBA? To better grasp the implementation of PIBA in CAP, the study is based on the framework of Normalization Process Theory (NPT). In this context, NPT (14, 15) offers a model consisting of four core components (16): (1) *Coherence* (the sense-making of staff both individually and collectively when faced with operationalizing PIBA in their units), (2) *Cognitive Participation* (the relational work staff do to build and sustain a community of practice around PIBA), (3) *Collective Action* (the work carried out by staff to enact and implement PIBA) and (4) *Reflexive Monitoring* (explores the appraisal work of staff to assess and understand the ways that PIBA affect them and the adolescents). By primarily focusing on individual and collective behavior, these components aim to help us to understand implementation and, above all, to normalize new interventions in clinical settings. Since implementing PIBA in CAP may be a way of promoting epistemic justice and recovery (13) it is described as crucial in order to grasp the underlying factors that either facilitate or complicate this process, which is why implementation theories become useful when trying to describe the clinical work performed by staff.

The framework of epistemic injustice (17, 18) has been used to further deepen the understanding of the experiences of staff as well as to explore how they might support adolescents as epistemic subjects in defining their need for inpatient care. According to Fricker (17), epistemic injustice, and especially testimonial injustice, implies that someone is wronged in their capacity as a knower. Also, being subject to this is argued to be largely related to how different attitudes and perceptions are constructed concerning the social category to which a person is considered to belong. Certain categories tend to be more easily exposed to injustice than others, such as women, ethnic minorities and individuals in institutional care (10, 17). These categories often contribute to trivialized narratives, thus making a high degree of credibility impossible, while the notion that mental health problems complicate rational thinking makes it easier for epistemic injustices to become self-generating. The body of research on epistemic injustice is growing, and more attention is being paid to children and young people (19–22), and addresses the paternalistic view of how the care apparatus defines what is in the “best interests” of the patients (23) as well as the general absence of epistemic subjectivity and lack of a co-creative climate in an individual’s encounter with care. What is also addressed is how the stereotypical image of mental health problems may undermine the self-knowledge of adolescents and diminish their capacity as knowledge bearers [cf. (24, 25)], which is why the field of epistemic research may be relevant when young people as epistemic subjects are examined in greater detail. Hermeneutic injustice is another aspect of epistemic injustice, meaning that someone’s ability to understand their (social) experience is hindered due to biases in our shared resources for social interpretation. According to Fricker (17), hermeneutic injustice is the injustice of having some significant area of one’s social experiences obscured from collective understanding owing to a structural identity prejudice in the collective hermeneutical resource. When exploring staff’s collective experiences of implementing PIBA, this approach might be helpful when trying to understand their reasoning and certain strategies to manage the practical implementation work.

### PIBA—From adult psychiatry to the context of adolescents

When trying to modernize the psychiatric system, it has been deemed necessary to incorporate new ways of promoting a more constructive care environment, where methods are continuously developed to optimize co-creation and participation. PIBA is described as a way of working with more complex mental health problems and is aimed at patients who have generally had a low degree of agency and self-determination in care. Since 2014, PIBA has been offered at several adult psychiatric clinics in Sweden. It initially started as a project addressing three different diagnostic groups—psychosis (26), emotional instability with self-harming and suicidal behavior (27) and eating disorders (28). In adult psychiatry, research shows coherent results regarding increased satisfaction and agency, fewer care days, increased suicide prevention work, reduced coercion in inpatient care and an improved care climate (29–32). These patterns of overall increased patient benefits may correlate with a recovery-oriented and person-centered approach (33–37). During the autumn of 2019, a political decision was made to implement PIBA in CAP in Stockholm with the ambition of increasing patient participation and agency among adolescents. Since December 2020, PIBA is offered to patients who meet the inclusion criteria, namely adolescents between 13 and 17 with an extensive need for care where more complex ill health, for example, self-harm, emotional instability, psychosis, and suicidal behavior, is common. Apart from parental consent, other prerequisites for receiving PIBA is that the adolescent has been admitted to inpatient care in the past year and has an expected great need for care ahead. Also, the adolescent needs to express his or her own desire to receive PIBA as well as demonstrate an understanding of the meaning of using it.

### Setting—The context of CAP

The units in focus in this article are part of a cohesive child and adolescent psychiatric organization. There is a total of 13 local outpatient units, eight outpatient units with targeted interventions around, for example, trauma, emotional instability, and psychosis, an emergency room and three inpatient units with 10 beds in each. Most of the admissions are described as voluntary, even though inpatient care also cares for young people against their will, according to the Compulsory Mental Care Act (38). The need for inpatient care is described as being greater than what the places can cater for (39), and in this context, PIBA is thus viewed as fulfilling an important function regarding accessibility when an adolescent deems that an admission is necessary. PIBA currently affects four outpatient units and one inpatient unit and thus concerns about 130 employees. At each unit, a designated nurse is appointed who is expected to have an insight into the implementation process, while all staff must know the basics of PIBA and have completed a web-based staff training course to ensure this specific knowledge.

## Materials and methods

As we wanted to investigate the staff’s joint understanding of PIBA, it seemed appropriate to conduct focus group interviews to obtain this specific knowledge since it is valuable tool for collecting qualitative data (40). Altogether, five focus group interviews were conducted, one in each participating unit. Four of them were conducted in the outpatient units by the first author (JM). The second author (U-KS) conducted one focus group interview in an inpatient unit and an additional individual interview with a person in managerial position, who expressed a wish for this. By using a semi-structured interview guide inspired by the four core components of NPT (16) concerning the implementation process, the ambition was to follow the reasoning of the staff. The guide included questions such as “How would you describe PIBA?,” “What are the prerequisites for implementing PIBA?” and “How does PIBA differ from regular work?.” All the interviews were held at the five units between December 2021 and April 2022 and lasted between 35 and 80 min.

### Description of the participants

In all, 27 interviewees, 21 women and six men, participated. They were recruited *via* the manager at each respective unit, and in each focus group there was a mixture of different professions such as unit managers, nurses, psychologists, care workers, counselors, and psychiatrists. Two of the nurses were also the designated contact persons for the implementation of PIBA.

### Analysis

After transcribing the audio-recorded interviews verbatim, the material was read through to obtain an overall picture of the content. The coding of essential content, particularly touching on descriptions of the conditions for implementing PIBA and how this work has been carried out in CAP, was performed by the first author. After scrutinizing the transcripts, the material was categorized meaning that adequate units were selected, condensed, and analyzed iteratively as themes and sub-themes emerged in accordance with a thematic content analysis (41) influenced by the four core principles of NPT (16). After that, discussions were held with the second author until a consensus on the themes was reached. Further, to better understand aspects of knowledge and power shown in the material, the framework of epistemic injustice was used to deepen the analysis.

### Ethical considerations

This study was granted ethical approval (Dnr: 2021-02790). All participants were given oral and written information about the study prior to the interviews. Informed consent was collected in connection with the interviews, and participants were told that they could decline to answer questions or leave the interview context at any time.

## Results

An analysis of the material revealed two main themes (see Table 1) and the following results are presented for each theme separately. Although these themes have different meanings, they are nevertheless intertwined to some extent. Important aspects are highlighted through a number of quotes followed by numbers that refer to specific focus groups. When a quote from the individual interview is used, the quote is, for ethical reasons, cited as belonging to the focus group made in the same unit.

**TABLE 1:** Themes and subthemes.

The staff’s experiences of PIBA	Managing clinical PIBA work
Understandings of the purpose of PIBA	Organizational readiness—Preparing for PIBA
A shared responsibility	From theory to practice—The importance of communication and collaboration
	Practical obstacles and ambiguities
	Implementing PIBA—“Just do it”?

## The staff’s experiences of PIBA

The interviews were largely characterized by discussions concerning the introduction of PIBA, and are here related to the different core components of NPT as well as aspects of power and agency addressed by the framework of epistemic injustice.

### Understandings of the purpose of PIBA

A majority of the respondents expressed a coherent view of how PIBA matches the overall organization, although outpatient care portrayed PIBA in a more positive manner which was contrasted by inpatient care who more clearly discussed challenges with the implementation. PIBA was generally described as a complement to existing care and not as a solitary intervention, and using PIBA for preventive purposes where the contract enables faster access to inpatient care, reduces assessments in the emergency room and decreases destructiveness among adolescents was discussed in all interviews. Staff viewed the reduction of assessments prior to admission as something that promotes the agency of adolescents. In addition, avoiding acute phases in their mental health status was regarded as one of the basic principles, while the knowledge that inpatient care is within reach may contribute to increased endurance in an adolescent who is battling against poor mental health. Not having to persuade healthcare services that admission is necessary, rather than having to signal ill health in various destructive ways, was declared as one important aspect of using PIBA which connotes increased agency in a person’s encounter with care.

I think that it’s an effective way of asking for help and support/and that it’s a very…functional alternative to becoming destructive. This is how I think it can best be used. That instead of self-harming or threatening suicide…it’s about “I need support *now*”. And then you get it. (FG1)

However, depending on whether the interviews were conducted with staff in either outpatient or inpatient care, there were differences among the participants, consequently leading to an expression of uncertainty about the purpose. The perceptions of the staff were influenced by how long they had worked in psychiatry and their previous experience of the relevant target groups for PIBA in CAP. The work was more readily perceived as futile as there was no coherent understanding of the purpose of offering PIBA, or where it was not known who had the overarching responsibility.

So, it’s very vague, and I think it’s because…we don’t really know the purpose. Honestly, I’d say we don’t know what we’re doing here. Is it suicide prevention? Who assesses those situations? (FG5)

The staff discussed PIBA in relation to regular practice, where a more accessible inpatient care was understood to be an important aspect for the adolescents. An extension of this discussion included PIBA being seen as a promising tool to promote knowledge justice and recovery and how this is best utilized in practice. A number of respondents emphasized that one of the main benefits of having a contract is knowing that care is within reach, which may help curb admissions. For example, it was often stated that it is sometimes sufficient for adolescents to call the inpatient unit to “check” whether there is a vacant bed. According to the staff, this aspect has an important preventive function in itself without adolescents actually “using” PIBA.

I think that PIBA…that its absolutely most important purpose is being an asset that you can reflect upon as a patient. You may not necessarily actually *use* it, but just as we have our telephone hotline, I think that PIBA is exactly the same type of experience for the patient…that it gives them a sense of security knowing that it’s within reach. (FG1)

By implementing PIBA, the idea of exaggerating various destructive behaviors may thus be reduced which, in addition to an increased quality of life and control for adolescents, was said to benefit the entire CAP in terms of assurance that care is available in a more unconditional way. From an epistemic justice point of view, the respondents also emphasized how important being believed is for adolescents for them to be able to take that crucial step and ask for help when they consider that they are in need of it.

It makes a huge difference when you’re in the critical situation, you’re not called into question and…and don’t have to fight for someone to believe in you. (FG2)

Respondents had different views concerning their introduction to PIBA. Some remembered exactly in which context or by whom PIBA was first presented. Others described how they are generally flooded with information from different sources, which is why their introduction to PIBA was generally perceived as unclear. Some said that they had probably heard about the implementation at a workplace meeting while others thought they had first heard about it during a lecture and a few even said that they had not heard about it at all. In terms of cognitive participation, some of the staff explained that a lack of resources affected their ability to familiarize themselves with what they were supposed to do. This understanding permeated both outpatient and inpatient care where staff perceived the decision to initiate the implementation as unclear.

It hasn’t been that…instructive at all. I’d say that it hasn’t been clear between managers here…and then you don’t really know what the purpose is. (FG5)

The decision to implement PIBA was depicted as being sanctioned on a political level, and a common understanding was that the politicians were eager for the work to begin as soon as possible, or as one respondent put it, “it became damn urgent”. Among staff in outpatient care, PIBA became an explicit tool to use in their work with adolescents.

It was more imposed on them (inpatient care) as an…an extra thing on top of the tough job they’re already doing…while it was more like an offer for us, and we just said “wow, this is great!” (FG1)

Thus, the implementation decision was made at a high level without the presence of the clinical staff, and this was generally perceived as having an impact on the employees’ attitude to PIBA. As the quote above also shows, a number of the respondents underlined that this decision was “imposed” on inpatient care, where hesitation and resistance arose while also affecting the overall pace of the implementation process. At the same time, the staff felt that there was an expectation to quickly operationalize the political decision, which resulted in a lack of both structure and ownership of PIBA.

I think that a certain organizational resistance is based on the lack of knowledge and that it’s something that’s just been ‘thrown’ at us. (FG5)

### A shared responsibility

Although staff described the adolescents as the actual users of PIBA, the staff are themselves users of the method, which here addresses strategies for implementing PIBA and what becomes important to them during this work. During the interviews, it appeared essential to strive for a unified view of which adolescents that are eligible for PIBA to better understand how the staff should act in accordance with this new way of working. When discussing the target group for PIBA, the general view was that it was aimed at adolescents between 13 and 17 with rather complex mental health problems such as self-harming behavior, suicide attempts, emotional instability, and psychotic episodes. However, this understanding was not evident among staff in inpatient care, who requested clarifications about the adolescents that might be relevant for PIBA. In terms of identifying adolescents, the staff called for a joint effort rather than it being imposed on specific individuals. In addition, the fact that they did not wish to be alone in this work was mainly about protecting themselves from different self-destructive behavior which was described as sometimes occurring in contact with the adolescents. Drawing on this, the discussion then dwelled on the actual responsibility placed on an adolescent through a PIBA agreement. The staff emphasized in particular an adolescent’s actual ability to make such a decision for him or herself in a situation where the adolescent needs help with their mental state, and that this process is largely related to age, maturity and acquired psychoeducation. By increasing the say that adolescents have in these decisions, it may be understood that the staff consider them as epistemic subjects rather than merely care recipients. However, there were concerns that, at too young an age, you cannot be expected to shoulder the responsibility that is required, resulting in a “conclusion” regarding who is best suited for PIBA.

The optimal PIBA-patient is someone who already works with anxiety management, such as a DBT patient who’s over 15/who’s already been diagnosed./Someone who…can work with skills and has started with it and who wants to test the skills they’ve already acquired. (FG5)

Here, it seemed important to offer PIBA to motivated and determined adolescents who may use it to curb a deterioration in their mental health in time, rather than succumb to destructiveness. However, in relation to certain diagnoses, one respondent expressed the following:

I’d say that the ‘perfect patient’ is someone who’s…motivated. Those that know they want…to fight for their mental health. Um…I don’t think that a certain diagnosis is relevant. It’s mostly about…having to want it yourself…because we can’t force anyone to use PIBA. It has to be a choice made by the patient. (FG5)

When discussing the advantages of PIBA, discussions about the disadvantages and concerns about offering it also followed. These concerns were manifested in various ways, but mainly addressed the dynamics and overarching structure of inpatient care, where the possibility of promoting epistemic agency was described as limited with the risk of adolescents becoming hospitalized and subjected to epistemic injustice and further paternalism. During the interviews, inpatient care was claimed to be a temporary element in a person’s life, where “leaving” psychiatry is a goal in itself. Due to this, PIBA becomes a strategy to remain in care which is unsettling, according to the staff. When talking about this, it was suggested that PIBA might risk strengthening the identity of an adolescent as a “patient” by facilitating admissions for certain adolescents who often have extensive experiences of institutional care. Using PIBA may thus contribute to prolonged care periods, which is something that the staff needs to take responsibility for and monitor together in the midst of the overall implementation.

Before admitting the patient…I think you should be vigilant about whether the patient risks hospitalization…that you identify patients who are at risk. In my experience, patients who are hospitalized begin their journey in inpatient care. And then they can’t or don’t want to be discharged…then the patient has become ‘addicted’ (to inpatient care). (FG3)

## Managing clinical PIBA work

Incorporating a new way of working into an already pressured organization was portrayed as a challenge by all the respondents, with an emphasis on the general lack of resources in CAP. During the interviews, the implementation of PIBA in practice was often touched upon and particularly prominent was how to manage the overall responsibility of taking the theoretical understanding of PIBA and incorporating it into the clinical setting.

### Organizational readiness—Preparing for PIBA

Preparing for PIBA was explained as an indispensable element in terms of creating procedures but also ensuring that the organizational changes permeate all levels of care in CAP. The perceived hasty political decision was described as decreasing the agency of the staff, which meant that they were not provided with the best conditions for preparing in a sufficient way. This was understood as having affected the stability of the implementation as well as the staff’s attitude to it. The training that the staff were expected to receive—watching a PIBA video, receiving information orally and being shown the designated bed—was explained as important, although the majority of the respondents did not have the time to participate in or complete it. Also, when preparing for PIBA, the staff did not consider it to be an adult model that could be applied to CAP without adaptations.

I think they’ve just tried to implement PIBA as it looks in adult psychiatry. Like ‘this is what it looks like in the adult world, let’s take it to CAP’. But then…you have to deal with parents (laughs), which becomes a completely different thing. So, I think you need to adapt the idea of PIBA for someone under 18. (FG5)

When trying to reach a consensus, the staff felt that they needed an established dialogue between outpatient and inpatient care. Some outpatient units described themselves as “ready” to implement PIBA, but that the inpatient care setting was prolonging the process. Inpatient care has had a number of challenges to deal with, for example, the structures for contract writing, securing training opportunities and keeping the PIBA bed vacant. At the same time, they are faced with high staff turnover and expectations of accessibility from adolescents, parents and other healthcare providers. Based on these conditions, the outpatient care staff can subsequently see that inpatient care would have needed more time to prepare *before* the outpatient units started the implementation.

This is a consequence of…them getting PIBA in their lap. Their structure for this hasn’t been clear…staff haven’t felt safe…um…they haven’t even…they don’t know how to write (the contract). And then this is what happens. (FG1)

### From theory to practice—The importance of communication and collaboration

Two different starting points for implementing PIBA emerged during the analysis. Since they were involved in the preparatory work, staff from outpatient care reasoned on a more theoretical level regarding how to put PIBA into practice. However, when speaking to the inpatient care staff, who provide the actual care, they, in turn, reasoned in a more practical way. Organizational affiliation may influence how your understanding of PIBA is formed, at the same time as practical conditions for the implementation affect the entire CAP with a certain focus on communication between outpatient and inpatient care.

You probably need to sort of overcome all the obstacles and see how you can solve them. Because as things stand right now, it’s all very unwieldy. We’ve had difficulties with communication. How should we communicate and with whom? Just sending information between unit managers and those with a responsibility for PIBA is a huge thing. There’s a lot that’s unclear. (FG5)

Lack of collaboration was thus explained as an obstacle to the implementation. Rather than being empowered by collective action and performing new and meaningful tasks, working with PIBA becomes something that needs to be balanced in the midst of managing ordinary working tasks. Feelings of inadequacy were said to affect the everyday management of staff and they also impinged the organizational attitude toward PIBA.

You have to do it in a different way, there must be another ‘setting’ to make people want to work with this./To get to the point where staff’s more likely to say: “we feel safe with this, we have a readiness to be able to take care of this.” (FG4)

### Practical obstacles and ambiguities

Discussions about practical obstacles regarding the implementation of PIBA permeated all the interviews. In particular, the staff highlighted that what they were unable to achieve in their preparatory work, has a clear impact on the continued implementation. However, the most pronounced obstacle was portrayed as the organizational confusion surrounding the PIBA bed in inpatient care. According to the staff, the hasty decision about implementation meant that CAP did not have time to map out or communicate where the bed would be situated, resulting in uncertainty and frustration.

There was a lot of ambiguity about this bed and which inpatient unit it belonged to. One time, I found out that we no longer had it (PIBA) but we had told the emergency room that we couldn’t enroll a patient because we didn’t have any room since the PIBA bed was supposed to be kept vacant. And then someone said “but you no longer have PIBA in your unit”…and I was like “oh, don’t we?” And a month later someone said “but now you have PIBA again” (laughs). So that’s how it’s been./Also, at first, a patient could come (to the unit) 24/7 and then it changed so that patients had to come before…8 p.m. and then it changed again to 7 p.m. but that information wasn’t communicated to the night nurses and…um…the patients didn’t find out so they would appear at about 11 p.m. and were then told that they didn’t have access to PIBA. So, this has been a process. (FG5)

The staff also highlighted aspects of trust in relation to what is to be expected from staff in inpatient care. Uncertainties have so far led to outpatient care not really knowing if someone actually engages with the adolescents when they choose to use PIBA. Offering PIBA to a young person, where planning and contract writing is done together with the guardians, means that the staff must be able to deliver on the promises that were given prior to admission. Advocating PIBA thus requires that various processes between enrollment and discharge continue and that access to inpatient care is guaranteed in order not to undermine the agency of an adolescent. These uncertainties risk affecting the staff’s attitude with the result that PIBA is sometimes not considered at all.

Unfortunately, I think that an obstacle is about…the very practical aspects that sometimes make me think ‘she can just as easily go to the emergency room’…because…this PIBA bed may not be guaranteed. I don’t really trust the organization surrounding PIBA…or the access to inpatient care. I feel that we may take another road. We’ll solve this in some way or another. (FG1)

During the implementation, the staff have had to deal with missing information, for example, the respondents sometimes described that decisions were being made without them being able to identify by whom—external supporter, unit manager, section management or at an even higher level. Also, the staff currently experience ambiguities about what is expected of them and who has what responsibility in outpatient and inpatient care. A majority of the respondents claimed that the division of responsibilities and cooperation was unclear, and that it is important to illustrate *how* adolescents are identified, *who* is able to identify them and *who* to turn to if you have questions.

If there’s a disagreement among colleagues about this (PIBA)/then it must be handled in some way…somehow you have to agree if a patient’s ‘ready’. And who has the decision-making power? Is it the patient’s main therapist or is it the doctor in the unit as well? (FG2)

Being in agreement was thus described as a prerequisite if PIBA is to function as favorably as possible, which addresses aspects of collective action as well as the power and hierarchy structure in CAP. Not being synchronized in this endeavor was described as undermining the stability of the implementation process as well as complicating the role of the contact persons for PIBA. Due to staff turnover, the stability that needs to exist around these staff members was said to be absent which is problematic since the contact persons are expected to participate at the meeting where the contract between adolescents, guardians and CAP is drawn up. Without this supportive function, the process is perceived as even more unclear and risks not being carried out.

This makes me feel unsure. I have five patients who should be called to this meeting, but… I’ve mixed emotions about that (laughs). It has to be good for the patients./Um…and above all this meeting has to be actually carried out…because they’re waiting and wondering “when will it take place?”. We haven’t received any feedback from any of them and they (inpatient care) don’t know when it’ll be…when this meeting can…take place. (FG5)

Also, the family’s involvement in PIBA was explained as a complicated aspect during the implementation, where the overall organizational challenges have not helped. The staff described a situation where there was a conflict between parents and colleagues when trying to clarify *who* wanted to use PIBA—a decisive factor since the adolescent’s agency is expected to control this. If there is uncertainty about how and by whom PIBA is utilized, the staff argued that there are no resources to respond to different wishes or handle complex situations when the adolescents come to the inpatient unit.

Sometimes, the parents complicate PIBA admissions, and patients have told me that they came here (to the inpatient unit) only because their parents ‘said so’. But also, sometimes parents come here signaling chaos which makes everything quite distorted. Whilst parents say they want their child to be admitted, the patient shows great reluctance shouting “I don’t want to be here”…which makes PIBA impossible. So, in those cases it turns into a matter for the social services. (FG5)

### Implementing PIBA—“Just do it”?

Initially, some respondents thought that implementing PIBA would be fairly straightforward. However, the lack of organizational readiness has led to a re-evaluation concerning this. A number of participants described a general motivation to “just do it” but that the commitment needed from both outpatient and inpatient care has not been established. To avoid stagnation, the staff discussed the importance of keeping on trying rather than waiting for the best conditions, and if PIBA is really incorporated in the various units, there are also opportunities to address the management regarding practical difficulties.

I think we just need to ‘start doing’ it and not be afraid and not…not think so much/and if we just do it, we can also give feedback to managers that ‘this is how you could do’ or ‘this doesn’t work’ or ‘we need *this* to make it work.’ (FG3)

Going forward, it was considered crucial to have regular follow-ups and reminders about current adolescents, the criteria that apply to obtain an agreement and who the facilitator in each unit is, which constitutes reflexive monitoring. Since the implementation is characterized by a general confusion and uncertainty, not discussing PIBA often enough may, according to the staff, lead to PIBA being abandoned. Jointly monitoring the purpose and outcome of PIBA was claimed to be important since epistemic agency is not something that comes automatically by just implementing a method. Rather, this effort needs organizational and individual supervision, and being reminded of this at workplace meetings makes it possible to alleviate the risk of misunderstandings.

There has to be someone who has control…like ‘these are the criteria for PIBA, this is how you do it’, um…because otherwise it’s easy to forget. Sometimes we sit at treatment conferences and wonder ‘how do we do this?’. And then you don’t really know what to do because you don’t do it (discuss PIBA) often enough. (FG2)

Among the outpatient care staff, the idea of “just doing it” was also translated into the overall importance of trying new things and seeing the early implementation phase as work in progress. At the same time, the importance of having a committed management that is continuously involved in the implementation at the same time as they have the ultimate responsibility for the monitoring of general progress concerning implementation was accentuated. Without a joint organizational approach and clear directives in the process going forward, the work will be made more difficult, according to the staff.

If you’re going to implement this, it’s important that you’re…that the entire clinic, right from the top… has the will. That ‘this is what we’re going to do.’ And that it’s also communicated to the emergency room and chief physician and…everywhere. And you have to work with that for quite some time before it settles. (FG5)

## Discussion

The aim of this study was to explore staff’s perspectives of PIBA for adolescents with complex mental health problems, and what facilitates or hinders its implementation. In semi-structured focus group interviews, outpatient and inpatient care staff shared these experiences, and a number of dominant themes have been identified and interpreted within the theoretical frameworks of epistemic injustice and NPT.

### Conditions for implementing PIBA

When discussing the organizational conditions required for the implementation of PIBA, they were mainly described as insufficient which has contributed to the purpose of PIBA being perceived as fragmented. In CAP, the structure of the implementation has changed repeatedly, which the participants described as aggravating in terms of uncertainty and frustration. These factors risk affecting their view of PIBA as complicated, resulting in an incomplete or protracted normalization [cf. (14, 15)] meaning that important health benefits among adolescents here risk being lost [cf. (42)]. Basic prerequisites in the implementation process were about commitment among staff, time to get acquainted with PIBA, cooperation between outpatient and inpatient care as well as continuity for the designated facilitators of PIBA and adequate procedures for the writing of contracts. The absence of this organizational foundation may affect the general attitude toward PIBA.

Apart from the practical obstacles experienced by staff, the interviews also touched upon trust, professional expertise and leaning on each other’s knowledge as well as stable care chains when facilitating PIBA in the clinical setting. These conditions were said to be imperative in order to ensure organizational cohesiveness and readiness as well as promoting the continued implementation and epistemic agency among adolescents. Linking this to implementation theories, NPT addresses how different components in this process are approached by clinicians as well as how these are adopted in existing procedures. It also presents facilitating and hindering factors for this endeavor in that it stresses that this new way of working needs to be adopted correctly (43) where a joint organizational approach requires careful planning with an understanding of each other’s clinical everyday life. Here, staff presented different obstacles when working with PIBA—rather than relying on each other’s knowledge in offering PIBA to adolescents, ambiguities such as not knowing the facilitators or which inpatient care unit is responsible for the PIBA bed were described as barriers to PIBA being used or considered. This might complicate the above-mentioned core components of NPT, which is a finding that is in concordance with previous research illustrating the importance of stability and an overarching commitment when initiating implementation work [cf. (44)].

Furthermore, since the decision to implement PIBA was sanctioned on a political level, the way the staff reasoned regarding this can be understood on the basis of both epistemic injustice and implementation theories. As mentioned above, PIBA has the potential to promote epistemic justice for adolescents by allowing them to themselves define their need of inpatient care. Another result is that staff, especially in inpatient care, are also actors in this process but that they, to some extent, lack hermeneutic justice where they describe that their needs and wishes regarding the implementation work are diminished and/or made invisible. In terms of epistemic injustice, the staff had limited access to information, resulting in loss of knowledge, and power [cf. (17)]. Also, their agency was virtually non-existent and they did not participate in shaping how the work with PIBA was to be realized in practice. This is particularly addressed by the staff in the results section when they discuss the feeling of having PIBA “thrown” at them, meaning that they feel that the political decision was “top down” (45), [cf. (44, 46)] because staff were not involved in the different decision-making processes. Trying to express an opinion on the clinical work—such as adapting PIBA from a system for adults to one for adolescents and voicing their collective dissatisfaction with the organizational conditions—one way of increasing the staff’s agency and enhancing recovery orientation (47), is to reduce hermeneutic injustice (17) by recognizing staff as knowledge carriers as well as to underline the need for a “bottom up” perspective [cf. (44)]. However, it was implied that staff were not listened to when trying to voice dissatisfaction or concerns regarding practical matters and ambiguities during the implementation, and without acknowledging the coexistence of multiple perspectives concerning PIBA, for example, those of politicians, management and staff, the implementation risks failing due to resistance or abandonment which underlines the need for adopting both top-down and bottom-up approaches in order to facilitate normalization [cf. (43, 46)].

### Adjusting PIBA to the youth context

In order for staff to facilitate agency and recovery among adolescents, it was considered important to adapt PIBA from the adult setting and thus make adjustments to the youth context [cf. (13)] that includes parents. Part of the recovery research on young people is about how care can be optimized and developed to best facilitate a recovery-oriented approach (33, 37). Here, a “youth-friendly” perspective (35, 36) is argued to be established in an overall biomedically dominated range of care, where PIBA has the potential to realize this view as well as becoming an extension of young people’s right to agency and participation (1, 3–5, 34, 38). As an adolescent, being given the opportunity to use PIBA may be associated with increased psychoeducation which also stresses the general differences between adults and young people’s identity development. This developmental process usually includes aspects of ambivalence and uncertainty that need to be taken seriously in young people’s encounters with care [cf. (37)].

The involvement of parents at the admissions stage as well as transparency regarding how the adolescents use the contract were discussed by the staff, who wished for more adequate cooperation between CAP, adolescents and parents in order for PIBA to function optimally. Unlike adults who, may, without argument, renounce contact with family and relatives, adolescents were described as a part of the family system with a clearly limited legal space for self-determination [cf. (2)]. During the interviews, there was some uncertainty as to whether parents of adolescents with a PIBA contract risk persuading or otherwise influencing them to use it, which can understandably risk their agency and at the same time endanger their epistemic subjectivity. There were staff who had practical experience of having to manage the balancing act between, on the one hand, recognizing the young person’s increased agency in the choice to use the inpatient care and, on the other hand, not knowing of this choice being in accordance with what PIBA stands for, but rather a choice, formal or informal, made by the parents. In reality, this can mean that the adolescents are met by the staff’s confusion and uncertainty regarding the adolescents possibly being subject to parental guidance which may affect the admission in various ways, and especially the adolescents not being listened to [cf. (20, 24)] or seen as credible in their choice to use PIBA [cf. (21)].

### Methodological considerations

When interpreting these findings, certain methodological considerations need to be discussed. The presence of a unit manager in four of the five units may have affected the other participants. A limitation may be linked to the (potentially) reduced freedom that comes with a manager’s presence. Although in this context, the advantages have outweighed the disadvantages, especially as regards the possible uncertainties that may be clarified by people in managerial positions. A majority of the participants were from outpatient care, which means that the material and quotations may be perceived as uneven, since the perspective of inpatient care staff consists of a smaller sample. In light of the organizational limitations and differences in the work intensity between outpatient and inpatient care, the inpatient care staff were not interviewed as easily as the other staff. However, considerations were made that it was important to include the perspective of inpatient care as far as possible. Another reflection touches upon the fact that the units are in different phases regarding the implementation of PIBA, which may affect their understanding of and attitude toward the purpose of the method. Lastly, the main author has interpreted the material based on specific research questions linked to epistemic injustice and implementation theories, and thus omitted other possible themes than those presented here.

## Conclusion and implications for practice

This article reflects on the implementation of PIBA in CAP, and underlines normalization, epistemic agency and the position of adolescents in mental health care. The results of the study imply that the majority of the staff interviewed were positive toward the overarching ideas of PIBA and viewed it as a possibility to increase agency and recovery among adolescents and to legitimize their knowledge about their mental health status. However, the interviews also show that obstacles arise when work with PIBA is to be put into practice, where reduced agency among inpatient care staff may be a complicating factor for a sustainable implementation since they are expected to strengthen the agency and recovery of adolescents simultaneously. The results thus highlights the importance of promoting epistemic agency—such as organizational conditions and participation in the decision-making process—among the staff involved in implementing PIBA. Without these conditions, promoting epistemic agency and recovery among adolescents’ risks being reduced to merely a tokenistic vision rather than being properly put into practice. The paradigm shift toward recovery-oriented models in mental health care is ongoing and, to some extent, transcends CAP as well as other welfare services. Yet, since there is no sole manual for how agency and recovery are initiated and maintained, there needs to be a clearer understanding of what this entails when working with adolescents with complex mental health problems. Further, since it is not entirely obvious how the parents’ involvement should be shaped when PIBA is used, this calls for more scrutiny in order for PIBA to facilitate epistemic agency rather than hindering it due to parental involvement. In addition, to ensure that care is designed in a youth-friendly way, further work is required where above all the structural challenges of inpatient care are focused on. Moreover, while little attention has been paid to the experience of adolescents using PIBA, this should be the focus of future studies.

## Data availability statement

The datasets presented in this article are not readily available because participants of this study disagreed about their data being shared publicly. Requests to access the datasets should be directed to JM, jennie.moberg@socarb.su.se.

## Ethics statement

This study was reviewed and approved by the Swedish Ethical Review Authority (Dnr: 2021-02790). Written informed consent was obtained from the individual(s) for their participation in this study.

## Author contributions

JM elaborated the original theoretical proposal presented in this article and wrote the original manuscript. Both authors revised and edited the manuscript and were responsible for its final version.
